# Lidocaine infusions in chronic pain management: A prospective case series analysis

**DOI:** 10.1177/20494637211054198

**Published:** 2021-11-22

**Authors:** Elizabeth Vacher, Monika Kosela, Charlie Song-Smith, Fausto Morell-Ducos, Alan Fayaz

**Affiliations:** 1 Medical Students, 4919 University College London; 2 Anaesthesia and Pain Consultant, 8964 University College London Hospitals NHS Trust; 3 Honorary Lecturer, University College London; 4 Anaesthesia and Pain Consultant, University College London Hospitals NHS Trust; 5 Honorary Associate Professor, University College London; 6 Clinical Lead, Pain Education Research Centre PERC, University College London Hospitals NHS Trust

## Abstract

Chronic pain conditions are prevalent and cause a significant burden of disease. Intravenous lidocaine infusions have been reported to have an analgesic effect in patients with chronic neuropathic pain, but there is limited data supporting the efficacy of lidocaine across other chronic pain phenotypes. Our study aimed to evaluate the efficacy of a single infusion of intravenous lidocaine for pain relief and the impact on quality of life. We evaluated data from 74 patients with chronic pain who were treated with intravenous lidocaine at a specialist pain centre. Participants completed a questionnaire consisting of the Brief Pain Inventory (BPI) Short Form and additional EQ-5D quality of life metrics, before treatment and at follow-up.

Data comparing pain severity did not demonstrate a statistically significant change after treatment when averaged across the entire patient cohort (6.15–5.88, *p* = .106), irrespective of gender or pain phenotype. Scores for pain interference showed statistically significant reductions following treatment (7.05–6.41, *p* = .023), which may have been driven through improvements in sleep (7.41–6.35, *p* = .001); however, these reductions are not clinically significant.

The patient cohort was stratified into responders and non-responders based on >30% improvement in response to an overall impression of pain reduction question following treatment. In the ‘responder’ cohort, pain intensity scores showed a statistically significant reduction post-infusion (6.18–5.49, *p* = .0135), but no change was apparent for non-responders (6.07–6.09, *p* = .920). There were no differences between responders and non-responders for pain sub-types in our study.

This study found no difference in pain outcomes in a cohort of patients with chronic pain, a mean of 63 days following a single lidocaine infusion. However, a specific subgroup of responders may show slight improvements in some pain outcomes that may warrant further exploration.

## Introduction

Chronic pain, defined as pain persisting beyond the expected period of healing (in practice somewhere between 3 and 6 months) is a highly prevalent condition, estimated to affect between one-third and one-half of the UK population^1^ and is a major burden on healthcare services.^2^ Chronic pain may be considered a symptom of underlying disease or, in some cases, a disease in its own right (chronic primary pain).^3^ Chronic pain encompasses a wide range of diagnoses, which has important implications for management. Pharmacological treatment of chronic pain should take into consideration multiple factors, including the pain aetiology and patient characteristics. Impairment of mood, function and sleep are often reported in association with chronic pain, and the impact of treatments on these parameters should not be underestimated.

Lidocaine is an amide local anaesthetic, acting predominantly through blockade of sodium channels in the neuronal cell membrane, thereby reducing input from nociceptors – although pre-clinical studies also support an anti-inflammatory mode of action as well as potential central blocker of NMDA receptors.^4^ Lidocaine has been used in the management of pain in both acute peri-operative settings, as well as chronic neuropathic pain.^5–8^ However, evidence supporting its efficacy for chronic pain generally is limited. Lidocaine infusions were not included in the NICE 2021 guidelines for the management of chronic pain.^9^

Previous studies investigating lidocaine’s analgesic efficacy have been conducted in relatively small populations and typically reported on early (post-infusion) pain relief. Wren et al., 2019 reported a significant decrease in mean pain intensity scores following lidocaine infusions in a retrospective analysis of 40 patients with neuropathic pain.^10^ Pain was measured on a verbal numeric rating scale, with 0 indicating no pain and 10 indicating the worst pain imaginable, and the scores decreased from an average of 6.52 pre infusion to 3.19 post-infusion (95% Confidence Interval 1.88–4.79, *p* < .001).^10^ Another retrospective analysis of 74 patients with fibromyalgia who underwent three or more IV lidocaine infusions found that a longer-lasting effect was achieved with a dose of 7.5 compared to 5 mg/kg, although the primary outcome was derived from self-reported percentage pain relief only.^11^

Data from randomised controlled trials (RCT) investigating the utility of lidocaine for chronic pain have been mixed. In a 2019 trial including 34 patients with neuropathic pain, Moulin et al.^12^ found no long-term benefit in pain or quality of life compared to the control infusion. Another RCT including 42 neuropathic pain patients found multiple lidocaine infusions significantly reduced pain compared to placebo, especially after the third and fourth infusions, but this effect was not persistent at follow-up, 4 weeks post-infusion.^13^ Furthermore, a 2019 meta-analysis concluded that IV lidocaine is effective in relieving neuropathic pain immediately after treatment, but not over the course of weeks.^14^

Currently, there is sparse data on longer-term outcomes, of weeks to months, in patients with chronic pain conditions undergoing lidocaine treatment. Our study aims to evaluate whether a single IV lidocaine infusion offers sustained pain relief, and/or improvements in quality of life, in a population of patients with chronic pain. It is important to note that NICE guidance NG 193 ‘Chronic pain (primary and secondary) in over 16s’^15^ advises that intravenous local anaesthetics should not be offered for the treatment of primary chronic pain states outside the context of a clinical trial for complex regional pain syndrome. The data in this evaluation were collected well in advance of the publication of this guideline in April 2021. In addition, the guidance recommends that treatments that were initiated prior to the guideline publication should not be withdrawn if they are effective in helping patients manage their pain. We considered our study particularly apt in view of this guideline as it afforded an opportunity to evaluate the efficacy of these treatments where they were commenced prior to publication of the guideline.

## Methods

We performed a prospective case-series analysis of patients treated with a lidocaine infusion between June 2018 and July 2020 at specialist tertiary metropolitan hospital. The study was registered as a service evaluation with the local governance board and retrospective approval for publication of the data was sought from the trust Caldicott guardian. Ethical approval was not considered necessary after consultation with the online Health Research Authority decision tool.

### Patient cohort

The cohort was derived from adult patients with chronic pain, who had been referred for a trial of IV Lidocaine, following a consultation with a pain physician. Lidocaine was administered at a dose of 3 mg/kg at a rate of 1.5 mg/kg/h as an intravenous infusion over 2 h, in accordance with the local policy. Inclusion criteria for our analysis were first treatment with intravenous lidocaine occurring between June 2018 and July 2020 and completion of questionnaires at both baseline and follow-up time-points.

### Data collection

Data was collected by pain procedure nurses using pre-printed questionnaires and subsequently entered onto an electronic database, stored on the trust secure server. Data points included age, gender, pain location and current list of pain medications. Patients were also asked to complete Brief Pain Inventory (BPI) Short Form and EQ5D questionnaires in person, before their first infusion and at follow-up.

At follow-up, patients were asked to estimate perceived percentage pain relief from lidocaine infusion and report Patient Global Impression of Change (PGI-C) scores. The questionnaires were then repeated via phone within an average of 63 days (range 30–240) after their first lidocaine infusion (follow-up questionnaire). It is important to highlight that this significant range in time to follow-up may have impacted pain outcomes, since delays in outcome data collection may influence how pain outcomes are reported by patients, in addition to the intrinsic differences in lidocaine effectiveness over time. The decision to include all patients with complete follow-up data, irrespective of the latency to follow-up, was taken in order to maximise the study sample size.

Pain phenotypes were established through interrogation of patient records on the electronic health record system (EHRS) at the trust. Any incomplete data in the questionnaires was cross-referenced with EHRS.

### Outcomes

Average pain intensity on the BPI questionnaire (short form), calculated as the arithmetic mean of the four BPI pain intensity subdomains, was designated the primary outcome. Secondary outcomes included BPI pain interference scores, EQ-5D subdomains and patient impression of overall improvement in pain due to lidocaine. These outcomes were analysed for the main cohort of patients (*n* = 74) with further breakdown into groups based on pain phenotype.

In addition to the four individual BPI pain intensity subdomains, individual BPI interference scores were also analysed for the groups described above. The arithmetic mean of the following interference subdomains was also calculated to give an average pain interference score: interference with general activity, mood, walking ability, normal work, relations, sleep and enjoyment of life.

### Statistics

All data were analysed in GraphPad Prism (Version 9.0.2 for Mac OS). A t-test was applied to investigate the change in BPI scores pre and post infusion. The analysis was initially carried out for the full cohort, and subsequently for subgroups of patients according to gender and pain phenotypes, to see if different pain phenotypes responded differently to the infusions.

## Results

### Patient characteristics

**Figure 1.**
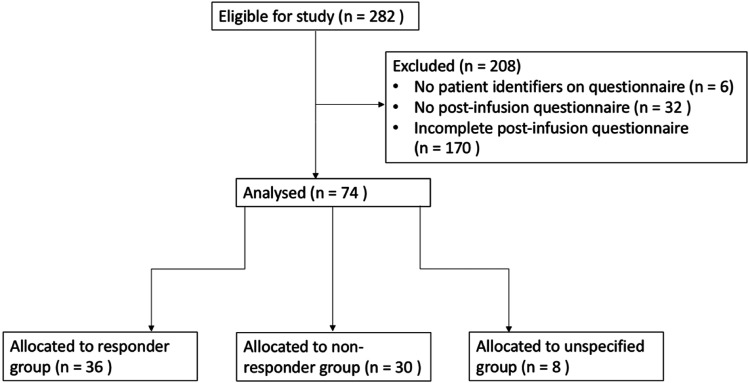
Diagram to show allocation of individuals in study.

282 patients received a lidocaine infusion between June 2018 and July 2020, and of these, 26% (*n* = 74) had complete pre- and post-infusion questionnaires (Figure 1) which fulfilled the primary outcome of change in BPI average pain score. This was due to phone-based follow-up proving ineffective at obtaining follow-up questionnaires from many patients. Hence, 74 participants met our inclusion criteria and were included in the study, as shown in Figure 1. Table 1 describes the patient demographics of the 74 patients that were included in the analysis. The baseline characteristics of those eligible for study, shown in Table 2, were similar to those in the analysis cohort.

**Table 1.** Patient demographics.

Patient Characteristics	
All patients (n)	282
Age, mean (range)	49.4 (19–87)
Sex, % female	208 (73.8%)

Note that totals do not add up to 100% due to some patients fulfilling inclusion criteria to more than one group.

**Table 2.** Baseline demographics of all patients receiving a lidocaine infusion between June 2018 and July 2020.

Pain Phenotype	No. of Patients	% Patients
NeP	32	43.24
APP	19	25.67
CWP and/or FM	19	25.67
NSLBP	8	12.16
Other	3	4.05

APP: abdomino-pelvic pain; CWP: chronic widespread pain; FM: fibromyalgia; NeP: neuropathic pain; NSLBP: non-specific low back or neck pain. Other diagnoses included headache (*N* = 1), inflammatory bowel syndrome (*N* = 1), and myofascial pain syndrome (*N* = 1).

The average age was 49 years (range 19–87) and 77% of patients were female, as shown in Table 1. The percentage of patients on specific pain medications at the time of their first lidocaine infusion is also shown in Table 1. Of the 74 patients, 42 (56.76%) had been prescribed anti-neuropathic medication and 32 (43.24%) opioid medication. Table 3 shows the distribution of pain phenotypes within the total cohort.

**Table 3.** Pain phenotypes within the patient cohort.

Patient Characteristics	
All patients (n)	74
Age, mean (range)	48.6 (19–87)
Sex, % female	57 (77.03%)
Baseline BPI average score	6.15
Baseline Interference scores’ mean	7.05
Medications	
NSAIDs, % patients	28 (37.84%)
Opioids, % patients	32 (43.24%)
Antineuropathics, % patients	42 (56.76%)
No medications, % patients	3 (2.7%)

NSAIDs: nonsteroidal anti-inflammatory drugs. Antineuropathic medications included gabapentinoids, TCAs, and SNRIs. Note that totals do not add up to 100% due to some patients fulfilling inclusion criteria to more than one group.

### Primary and secondary outcomes for main cohort

#### Pain intensity

Mean pain severity (the arithmetic mean of the BPI pain item scores) did not demonstrate a statistically significant change after treatment when looking at data across the entire cohort (6.15–5.88, *p* = .106), nor when patients were stratified according to gender or pain phenotype (see Appendix 1).

#### Pain interference

Mean pain interference scores were calculated as the arithmetic mean of the interference domain scores (a–g: general activity, mood, walking ability, normal work, relations, sleep and enjoyment of life). Mean pain interference was reduced following treatment (7.05–6.41, *p* = .023), which may have been driven through improvements in sleep (7.41–6.35, *p* = .001) (Figure 1) since this was the only reduction in an interference domain that was statistically significant within the individual domains averaged. It is unlikely that this reduction is clinically significant.

#### Quality of life

Across quality of life measures, individual EQ-5D measures showed no statistical significance, other than Usual Activities (3.40–3.1, *p* = .028) and EQ-5D Pain/Discomfort (3.72–3.39, *p* = .018).

### Responder analysis

**Figure 2.**
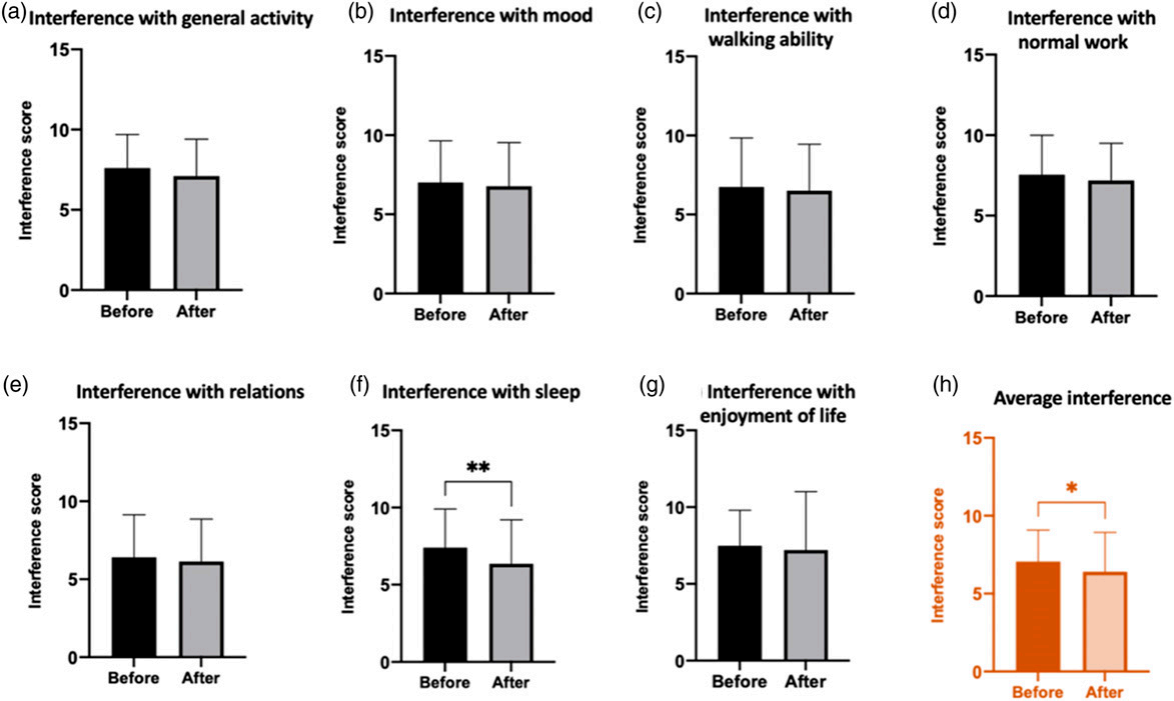
Average pain interference scores were reduced following treatment (7.05–6.41, *p* = .023), which may have been driven through improvements in sleep (7.41–6.35, *p* = .001).

The cohort was divided into responders and non-responders on the basis of self-reported overall impression of improvement in pain attributed to lidocaine infusions. This was determined by a specific question in the questionnaire that asked patients to quantify ‘overall pain relief from lidocaine infusion’, from 0 to 100% (Figure 2).

**Figure 3.**
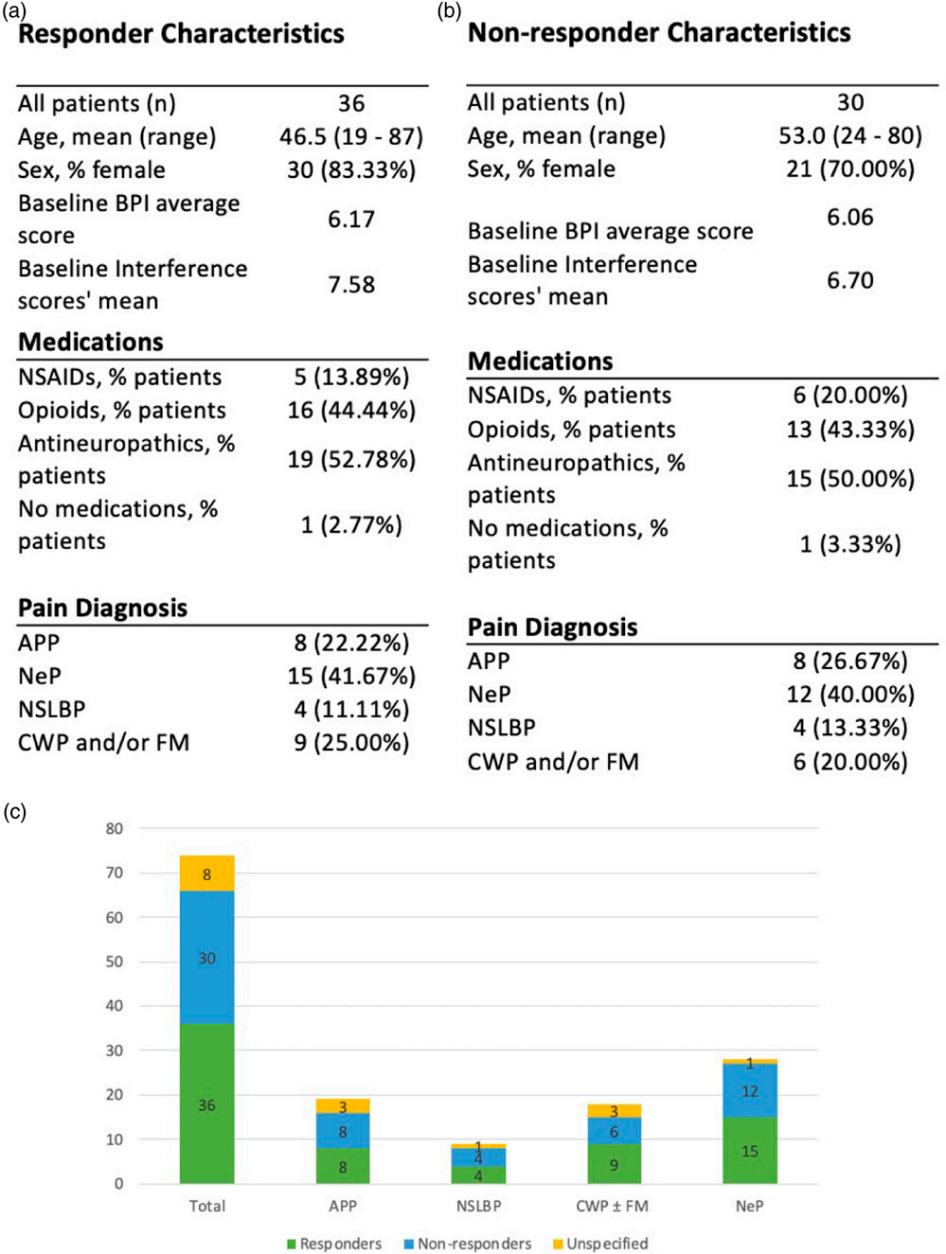
Comparison of responders and non-responders. (a) Responders’ demographics and pain phenotypes. (b) Non-responders’ demographics and pain phenotypes. (c) Proportion of responders and non-responders according to their corresponding pain phenotype. Note that totals do not add up to 100% due to some patients fulfilling inclusion criteria to more than one group.

Responders were defined as those who met a threshold of 30% pain relief in response to this self-reported impression of pain reduction. 36 of 74 patients (49%) reported 30% or greater subjective improvement in pain and were therefore included in the subsequent ‘responder analysis’. A comparison of the demographics of the responder group vs the non-responder group is shown in Figure 3. Women constituted 83.3% of responders (30 out of 36) and 70% of non-responders (21 out of 30). Men, respectively, accounted for 16.7% and 30% of responders and non-responders. The average age of responders was 46.5, while of non-responders 53. Eight of the 74 patients did not complete the question about ‘overall pain relief from lidocaine infusion’ and are therefore allocated to the ‘unspecified’ group (Figure 1).

The validity of the responder stratification was corroborated by scores on the seven-point PGI-C scale, which were significantly higher on average amongst responders than non-responders (4.8 vs 2.2, *p* < .0001). Figure 3 shows the baseline demographic and clinical characteristics of the responder and non-responder cohorts, as well as the distribution of pain phenotypes within these groups.

**Figure 4.**
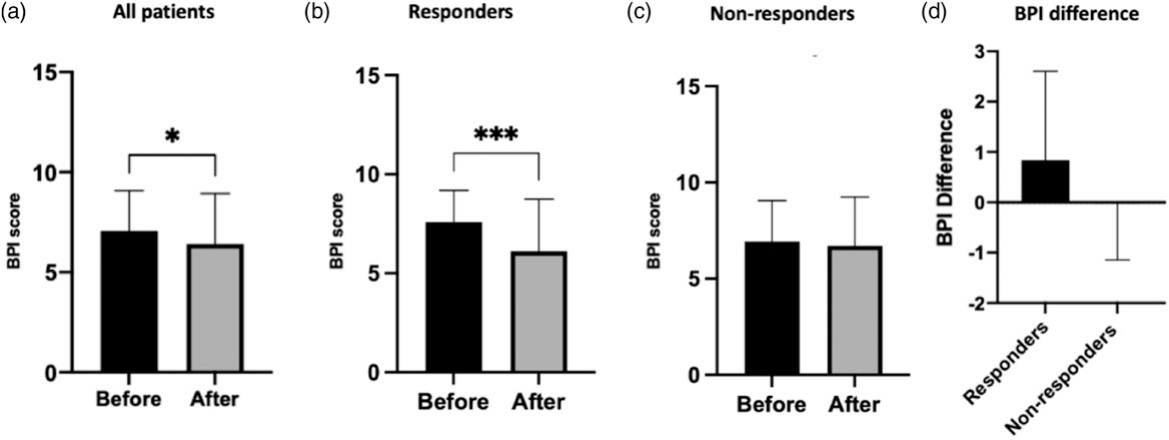
(a–c) Mean BPI Pain Severity Scores for all patients (6.15–5.87, *p* = .1060), responders (6.17–5.49, *p* = .0135) and non-responders (6.07–6.09, *p* = .9298). d) Difference in the mean BPI Pain Severity Score (before and after the first lidocaine infusion) was significantly different between responders (≥30% subjective pain relief following lidocaine infusion) and non-responders (.833–.026, *p* = .0257).

Paired t-test analyses were run, comparing the mean BPI pain severity and pain interference scores among responders and non-responders. BPI mean pain severity scores were significantly reduced post-infusion among responders (6.18–5.49, *p* = .0135) but not non-responders (6.07–6.09, *p* = .920) (Figure 4 (a)–(c)). In addition, the difference in the mean BPI pain severity score was significantly different between responders (≥30% subjective pain relief following lidocaine infusion) and non-responders (*p* = .0257) (Figure 4(d)).

**Figure 5.**
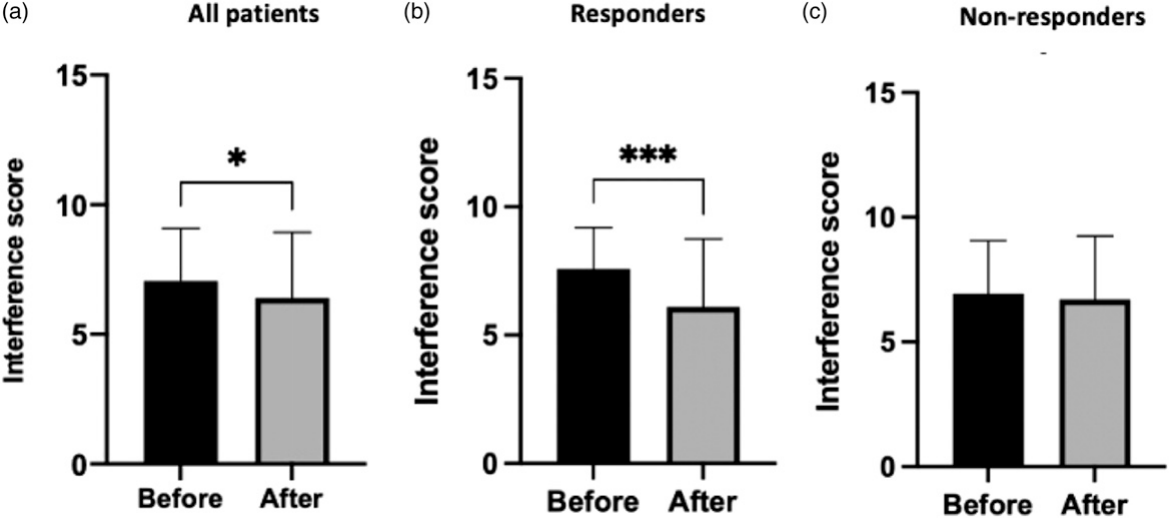
Mean BPI pain interference scores for all patients (7.05–6.41, *p* = .0226), responders (7.58–6.10, *p* = .0009) and non-responders (6.93–6.71, *p* = .38).

Comparison of BPI Pain Interference score (the mean of the seven BPI pain interference items) showed a statistically significant reduction in the mean pain interference score, following a lidocaine infusion, among all patients (7.05–6.41, *p* = .0226) and responders (7.58–6.10, *p* = .0009), as shown in Figure 5. In contrast, the average post-lidocaine pain interference score was not significantly altered among non-responders.

### Pain subgroup analyses

In addition to assessing any changes in BPI and EQ5D scores and subscores in the overall patient cohort, BPI subscores before and after lidocaine were compared within the pain subgroups of APP, NeP, NSLBP and CWP/FM.

**Figure 6.**
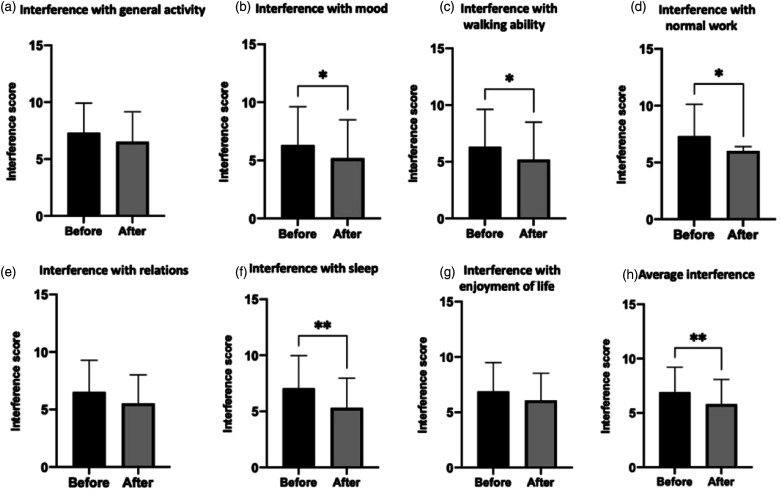
Mean Pain Interference Scores for APP subgroup, pain interference with mood (6.89–5.92, *p* = .047), walking ability (6.32–5.18, *p* = .016), normal work (7.32–6.05, *p* = .012), sleep (7.05–5.32) and mean interference (6.90–5.80, *p* = .008) all showed a statistically significant reduction.

In the APP subgroup, none of the BPI pain subscores showed a statistically significant difference after treatment, but pain interference with mood (6.89–5.92, *p* = .047), walking ability (6.32–5.18, *p* = .016), normal work (7.32–6.05, *p* =.012), sleep (7.05–5.32) and mean interference (6.90–5.80, *p* = .008) all showed a statistically significant decrease (Figure 6). Likewise in the NeP subgroup, no BPI pain severity subscores were significantly reduced, while interference with sleep was the only pain interference measure that showed a statistically significant reduction (7.41–6.35, *p* = .023). These reductions are unlikely to hold clinical significance.

No measures were significantly different in the NSLBP cohort, while BP pain at its worst in the last 24 h (7.79–7.03, *p* = .041) and pain on the average (6.58–5.90, *p* = .044) were the only measures that were significantly lower post-treatment amongst the CWP/FM subgroup.

## Discussion

In this prospective case series, the administration of a 3 mg/kg weight of IV lidocaine infusion was not shown to significantly improve pain or quality of life for a cohort of chronic pain patients. Average pain intensity (as measured by the mean BPI pain intensity score) was not significantly reduced amongst the study population. Although mean pain interference and specifically, sleep interference, showed a statistically significant reduction in the study population, it is unlikely that this may translate to a clinically significant reduction. Additionally, lidocaine’s effects on sleep have not previously been reported or formally investigated, so it is uncertain whether this finding is reproducible.

Within our study population, we identified a group who seemed to report more significant benefits in response to IV lidocaine infusions. These ‘responders’ were defined as those who self-reported a 30% or greater improvement in their pain due to lidocaine in response to a global impression of pain reduction question, and this was intended to mirror the NeuPsig guidelines for neuropathic pain.^16^ There is a rationale for identifying whether individual patients might respond to IV lidocaine infusions. Although we found no difference in response between chronic pain phenotypes, this may have been limited by the very small sample sizes of the pain phenotype subgroups. Whilst a placebo effect could be contributing to the reported 30% improvement in pain, the NeuPsig guidelines recommend that a 30% reduction is likely to be clinically important.^16^ As with the entire cohort, it is important to note that whilst responders demonstrated a statistically significant reduction in average BPI pain score and average pain interference, this may not have clinical significance. For example, the reduction in Mean BPI Pain Severity Scores was only 12% (6.17–5.49), well below the 30% stipulated by the NeuPsig guidelines.

Responders in our analysis were more likely to be female and younger on average than non-responders, but further research may be needed to identify the specific characteristics of patients that are more likely to respond to IV lidocaine infusions.

Our data add to the relatively small number of studies reporting pain-relief and quality of life outcomes beyond the immediate post-infusion period after treatment with intravenous lidocaine. This study differs from most of the existing literature and previous studies on lidocaine for chronic pain in several ways. We analysed data from patients with a variety of chronic pain phenotypes, including both neuropathic and non-neuropathic pain, whereas most studies investigating lidocaine treatment to-date have been limited to neuropathic pain patients. This heterogeneity may be considered a limitation due to the small population size of the study, since the study was not adequately powered to assess the utility of lidocaine for the specific pain phenotypes identified. While many studies have investigated the analgesic effects of lidocaine in the days and weeks after treatment, few have reported on longer-term outcomes. In contrast, in our study, patients were followed up a mean of 63 days after their first lidocaine infusion. However, it is important to recognise that there was a significant range in the time period over which the outcomes were collected. In cases where outcome collection was more delayed, self-reported pain outcomes may be less accurate.

In addition, whilst we collected data on concomitant and rescue pain medication use, including individual drugs and any dose changes, it is difficult to quantify and analyse differences in the use of these medications between patients before and after infusions. This is because the use of concomitant treatments is straightforward to measure in single-dose analgesic studies, but more difficult in studies that follow participants over many months. Although changes in the use of concomitant pain treatment can be used as an outcome measure, and scales and composite measures have been developed that combine rate of medication usage according to dosage and medication class, these data were collected in a narrative, free-form manner in the follow-up questionnaires in our evaluation and could therefore not be condensed into a single measure that lent itself to objective comparison between patient subgroups. Other confounding factors such as socioeconomic status and medical and psychiatric co-morbidities were not collected.

At present, the most comprehensive appraisal of the efficacy of intravenous lidocaine for short- and long-term relief of chronic pain is a 2019 meta-analysis by Zhu et al.,^14^ which quantitatively analysed pain reduction after lidocaine infusions for patients with neuropathic pain. The authors divided studies into groups assessing pain relief from lidocaine in the immediate post-transfusion period and over the longer-term, respectively, although it is not clear what criteria were used to define the ‘immediate’ period after treatment. They found that lidocaine demonstrated a clear and statistically significant analgesic effect compared to placebo in a pooled quantitative analysis of 500 patients. However, they did not find a statistically significant reduction in pain compared to placebo beyond the immediate post-infusion period, suggesting that the analgesic effects of lidocaine are relatively short-lived. This could account for our detection of modest improvements in pain and sleep, which were statistically but not clinically significant, representing the ‘tailing-off’ of lidocaine’s post-acute effects.

This study has several important limitations. It should be noted that the data from our cohort of 74 patients is not necessarily representative of all patients attending the pain management centre with chronic pain and may not be generalisable to patients with chronic pain in general. Due to incomplete questionnaires, we were only able to analyse 26% (74/282 patients) of our eligible sample. Furthermore, our responder analysis included only 12.7% (36/282) of the patients who received lidocaine treatment at the centre during the data collection period; therefore, further studies in larger patient populations are needed to guide clinical practice. For our subgroup analyses, which again included very small sample sizes and were therefore statistically underpowered, any conclusions should be taken with caution.

Since baseline questionnaires were administered in-person under the supervision of trained staff, it is possible that the use of telephone follow-up may have contributed to differences in the data collected between the two time points. This method of data collection also severely limited the study sample size, since the primary and secondary outcomes could not be calculated for patients who had any data that was missing or incorrectly filled in, even if only in one subdomain of the questionnaire. Finally, given that we only reported outcomes in patients with a baseline and a follow-up questionnaire completed, it is possible that those patients who did not benefit from the lidocaine treatment were more likely to be lost to follow-up.

## Conclusions

This study does not demonstrate a difference in pain outcomes following lidocaine infusions in a cohort of patients with chronic pain; however, specific subsets of patients may show improvements that need to be explored further. This study adds further evidence to the existing data suggesting that the analgesic effects of a single IV lidocaine infusion do not persist beyond the short-term, most likely the weeks immediately following treatment. Although lidocaine produced a statistically significant reduction in average pain interference, the clinical significance of these improvements is uncertain and likely to be small. Further studies may be warranted to establish whether the improvements reported here are of real clinical benefit, to identify the patients who are most likely to respond to lidocaine and show any longer-term benefits of lidocaine treatment.

## Appendix 1

(a) Among all patients, the average BPI score was reduced from 6.15 to 5.89 following a lidocaine infusion (*p* = .1060).

(b) The difference of the average BPI score before and after a lidocaine infusion between female (.35965) and male patients (.61765) was not statistically significant (*p* = .0638).

(c–e) Average BPI scores were not statistically different after lidocaine treatment amongst patients with APP (5.68–5.28, *p* = .11934), NeP (6.19–5.74, *p* = .28212), NSLBP (6.28–6.62, *p* = .437029), CWP and/or FM (6.09–5.80, *p* = .1038).
